# Macrophage- and pluripotent-like reparative Muse cells are unique endogenous stem cells distinct from other somatic stem cells

**DOI:** 10.3389/fbioe.2025.1553382

**Published:** 2025-03-27

**Authors:** Mari Dezawa

**Affiliations:** Department of Stem Cell Biology and Histology, Tohoku University Graduate School of Medicine, Sendai, Japan

**Keywords:** pluripotent, phagocytosis, sphingosine-1-phosphate, HLA, immunotolerance, clinical trials

## Abstract

Muse cells are endogenous reparative stem cells with dual characteristics: pluripotent-like and macrophage-like. They can be identified by the pluripotent surface marker stage-specific embryonic antigen-3-positive (SSEA-3 (+)) cells in the bone marrow, peripheral blood, and various organs, including the umbilical cord and amnion. Muse cells can differentiate into ectodermal, endodermal, and mesodermal lineage cells, self-renew, and selectively migrate to damaged sites by sensing one of the universal tissue damage signals, sphingosine-1-phosphate (S1P). At these sites, they phagocytose damaged/apoptotic cells and differentiate into the same cell type as the phagocytosed cells. In this manner, Muse cells replace damaged/apoptotic cells with healthy, functioning cells, thereby repairing tissues. Due to their specific immunosuppressive and immunotolerant mechanism, clinical trials have been conducted for acute myocardial infarction (AMI), subacute ischemic stroke, epidermolysis bullosa, amyotrophic lateral sclerosis (ALS), cervical spinal cord injury, neonatal hypoxic-ischemic encephalopathy (HIE), and COVID-19 acute respiratory distress syndrome. These trials involved the intravenous injection of ∼1.5 × 10^7^ donor Muse cells without human leukocyte antigen (HLA) matching or immunosuppressant treatment, and they demonstrated safety and therapeutic efficacy. Thus, donor Muse cell treatment does not require gene manipulation, differentiation induction, or surgical intervention. These unique characteristics distinguish Muse cells from other somatic stem cells, such as mesenchymal stem cells, VSEL stem cells, and marrow-isolated adult multi-lineage inducible (MIAMI) cells.

## Introduction

Multi-lineage differentiating stress-enduring (Muse) cells, already used in clinical trials, are unique endogenous reparative stem cells with dual characteristics: pluripotent-like and macrophage-like (Kuroda et al., 2010; Wakao et al., 2011). They are suggested to be constantly supplied from the bone marrow (BM) to every organ through circulation, phagocytose damaged/apoptotic cells as model cells in each organ, quickly differentiate into the same cell type as phagocytosed cells, and replace damaged/apoptotic cells with healthy, functional cells, thereby participating in daily tissue homeostasis (Kushida et al., 2018; Tanaka et al., 2018; Minatoguchi et al., 2024).

Muse cells are identified as pluripotent surface marker stage-specific embryonic antigen (SSEA)-3-positive cells in the BM (0.01%–0.03% of the mononuclear fraction), peripheral blood (PB) (0.01%–0.2%), and connective tissues of every organ, including the extraembryonic tissues, umbilical cord, and amnion (Dezawa, 2016; Leng et al., 2019; Sato et al., 2020; Aprile et al., 2021; Ogawa et al., 2022). The developmental origin of Muse cells has not yet been elucidated; however, since they have a reserve in the BM, share similarities with hematopoietic stem cells, are widely distributed in connective tissues, and are also present in extraembryonic tissues such as the umbilical cord and amnion, it is possible that Muse cells originate from a lineage similar to that of the extraembryonic mesoderm.

Consistent with their being endogenous to the body, they are non-tumorigenic but can be grown on a clinically relevant scale as their doubling time is ∼1.3 days per cell division, comparable to that of fibroblasts (Ogawa et al., 2022; Wakao et al., 2011; Kuroda et al., 2013; Ogura et al., 2014; Gimeno et al., 2017).

Since Muse cells have specific immunosuppressive and immunotolerance mechanisms, HLA-mismatched donor-Muse cells can be directly administered to patients without immunosuppressants (Minatoguchi et al., 2024). Clinical trials have been conducted for acute myocardial infarction (AMI), subacute ischemic stroke, epidermolysis bullosa, cervical spinal cord injury, neonatal hypoxic-ischemic encephalopathy (HIE), amyotrophic lateral sclerosis (ALS), and COVID-19 acute respiratory distress syndrome by intravenous administration of donor Muse cells without HLA matching or immunosuppressant treatment. Their safety and therapeutic effects were reported (Noda et al., 2020; Niizuma et al., 2023; Fujita et al., 2021a; Yamashita et al., 2023; Sato et al., 2024; Koda et al., 2024).

Along with the human BM, Muse cells were discovered in cultured mesenchymal stem cells (MSCs) and fibroblasts, accounting for several percent of the total population (Kuroda et al., 2010; Wakao et al., 2011; Kuroda et al., 2013). Therefore, Muse cells exhibit properties similar to MSCs, such as expressing MSC markers and exhibiting bystander effects. However, several crucial differences exist between Muse cells and non-Muse MSCs, as described in the following sections. Furthermore, the characteristics of Muse cells are distinct from very small embryonic-like (VSEL) stem cells, and marrow-isolated adult multi-lineage inducible (MIAMI) cells, and their intracellular molecular mechanisms to maintain pluripotency are also different from those in embryonic stem (ES) and induced pluripotent stem (iPS) cells (Wakao et al., 2011; Kucia et al., 2006; D'Ippolito et al., 2004; Kuroda et al., 2022). Although some aspects of Muse cells have not yet been fully elucidated, their known characteristics suggest that they are unique and distinct from existing stem cells. Given their numerous clinical benefits, Muse cells hold potential for various therapeutic applications in the future.

## Similarity of muse cells and monocytes/macrophages

### Morphological characteristics of cells

**FIGURE 1:**
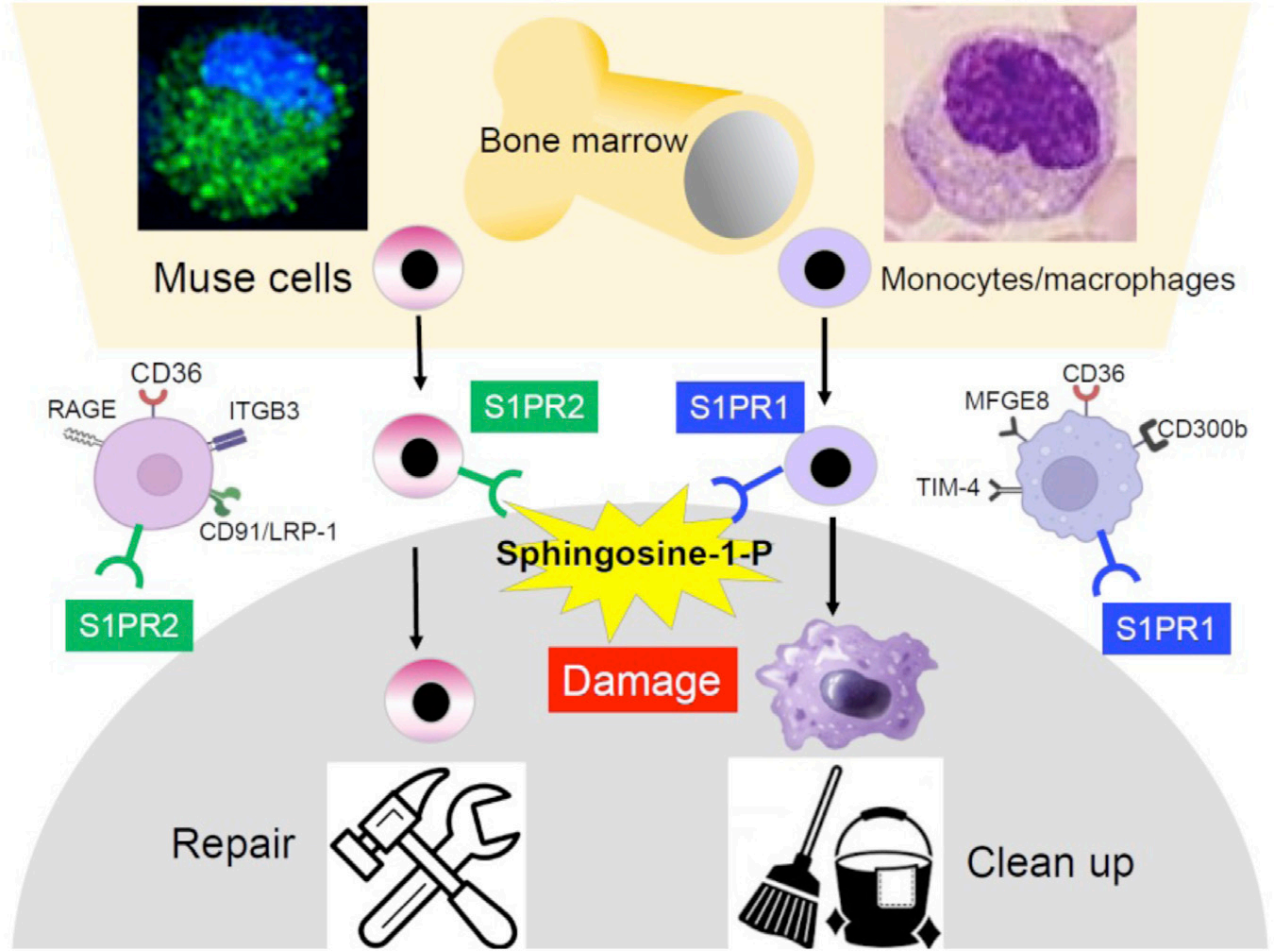
Similarity and difference between BM-Muse cells and monocytes/macrophages. Both are similar in the reserve site (the BM), size, morphology, response to S1P, phagocytosis-related receptor expression, and phagocytic activity but different in the S1P receptor subtype, repertoire of phagocytosis-related receptors, and differentiation ability. Monocytes/macrophages are professional phagocytes that clean apoptotic cells and cell debris, while Muse cells differentiate into the same cell type as the phagocytosed damaged/apoptotic cells.

Muse cells are similar to monocytes/macrophages in several points (Figure 1). The size of Muse cells in the PB and suspension culture is 10–15 µm in diameter with horse-shoe, beans, or heart-like nuclei, similar to monocytes in the PB (Sato et al., 2020). Muse cells in the PB are consistently SSEA-3 (+)/CD45 (+) cells (Sato et al., 2020).

### Expression of markers

Muse cells are contained in the mononuclear cell fraction of the PB. However, because of the similarity in size and shape, they are presumably counted among monocytes. In addition to the morphological similarity, Muse cells express monocyte/macrophage markers such as CD45, C–C motif chemokine ligand 2 (CCL2), CD80, interleukin-10 (IL10), interleukin-1 receptor 1 (IL1R1), and toll-like receptor 2 (TLR2) (Wakao et al., 2022). Muse cells in the blood express the pluripotency marker SSEA-3, and all of them also express CD45 (+) (Sato et al., 2020). On the other hand, Muse cells in the BM and connective tissues of each organ express SSEA-3 but do not express CD45 (Wakao et al., 2011). Muse cells in the blood and tissues are assumed to be continuous, and the microenvironment controls the presence or absence of CD45 expression. Still, the mechanism needs to be clarified in the future.

### Sphingosine-1-phosphate receptors

**FIGURE 2:**
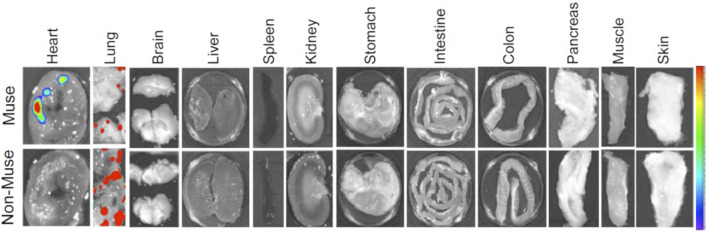
Selective migration of Muse cells to the site of damage following intravenous administration. A rabbit AMI model 3 days after intravenous injection of nano-lantern-Muse and -non-Muse MSCs exhibited specific homing of Muse cells to the post-infarct heart, while non-Muse cells were trapped in the lung and rarely homed to the heart tissue (Yamada et al., 2018).

Another similarity between Muse cells and monocytes/macrophages is that both selectively migrate to sites of damage in the body by sensing sphingosine-1-phosphate (S1P) produced by damaged/apoptotic cells (Wakao et al., 2022; Yamada et al., 2018). Responsiveness to S1P is sharp and swift in Muse cells and is driven by S1P receptor 2 expressed in Muse cells, while driven by S1P receptor 1 in monocytes/macrophages (Figure 1). Intravenously administered nano-lantern or Akaluc-labeled Muse cells selectively accumulated at the post-infarct area by day 1 in a lacuna stroke model and day 3 in an AMI model, showing their swift and accurate migration to the damaged site (Yamada et al., 2018; Abe et al., 2020; Figure 2).

### Phagocytosis receptors

**FIGURE 3:**
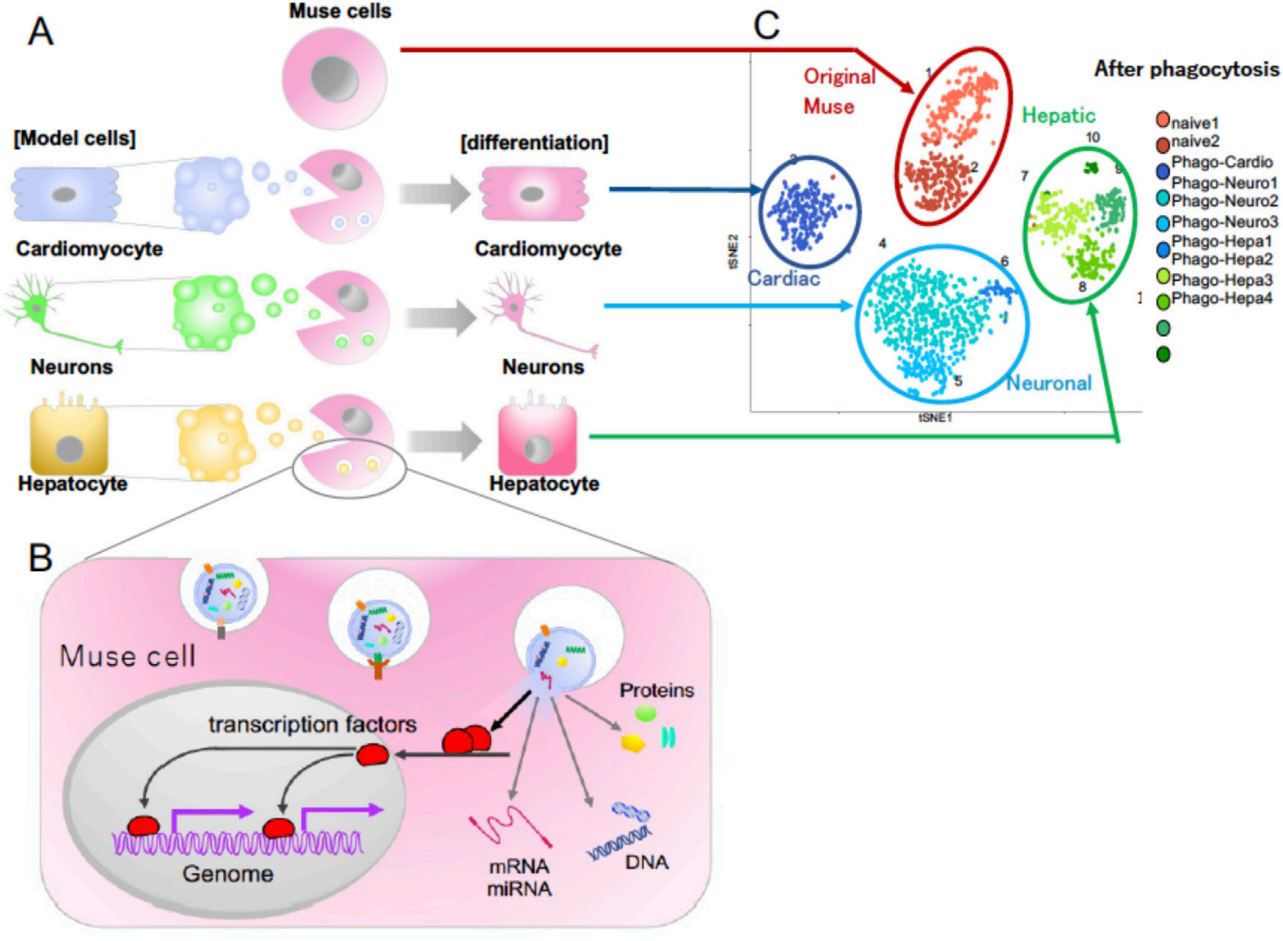
**(A)** Differentiation of Muse cells into the same cell type as the phagocytosed apoptotic cells. **(B)** Molecular mechanism of how Muse cells recycle signals from the up-taken damaged/apoptotic cells necessary for differentiation, such as transcription factors. **(C)** Single-cell RNA sequencing of human Muse cells (original Muse cells) after phagocytosing apoptotic cell fragments of mouse hepatic (Hepatic), mouse cardiac (Cardiac), and rat neural (Neuronal) cells at 7 days (Wakao et al., 2022).

The ability to perform phagocytosis, an outstanding characteristic of Muse cells, also suggests a similarity to monocytes/macrophages (Wakao et al., 2011). CD36 is commonly active in both Muse cells and monocytes/macrophages as a phagocytosis receptor. Still, other main receptors observed in Muse cells were ITGB3, CD91/LRP-1, and RAGE, which differ from those of macrophages, such as the TIM-4 family, CD300b, and MFGE8 (Wakao et al., 2011; Figure 1). Although the detailed repertoire of phagocytosis receptors differs between Muse cells and macrophages, both phagocytose damaged/apoptotic cells by sensing phosphatidylserine expressed on the cell surface. However, the changes in cells after phagocytosis significantly differ between the two. Monocytes/macrophages are professional phagocytes specializing in cleaning up; thus, the ingested cellular debris is quickly digested. On the other hand, although Muse cells contribute to cleaning to some extent, their primary mission is replacement through differentiation (Wakao et al., 2011; Figures 1, 3A).

### Phagocytosis-dependent differentiation in muse cells

Unlike macrophages, Muse cells directly recycle signals from the up-taken damaged/apoptotic cells necessary for differentiation, including transcription factors, and quickly differentiate into the same cell type as the phagocytosed damaged/apoptotic cells in days (Wakao et al., 2022; Figures 3A, B). As shown by single-cell RNA sequence analysis, there are few errors in the differentiation direction (Figure 3C). Phagocytosed cell-derived transcription factors translocate into the Muse cell nucleus after phagocytosis and bind to the promoter regions of the genomes, leading to the expression of lineage-specific markers corresponding to the phagocytosed damaged/apoptotic cell type within 15 h to several days (Wakao et al., 2022; Figure 3B). The phagocytosis-induced differentiation proceeds rapidly compared with *in vitro* cytokine-induced differentiation. Generally, cytokine- and/or gene introduction-dependent differentiation induction requires several weeks to months. In Muse cells, cytokine-induced *in vitro* differentiation into melanocytes, cardiomyocytes, and neural-lineage cells takes at least several weeks to months, with ∼80% of the cells differentiating into target cell types (Wakao et al., 2011; Tsuchiyama et al., 2013; Amin et al., 2018).

In this manner, Muse cells can replace various types of damaged/apoptotic cells with healthy, functioning cells in the tissue due to their pluripotent-like property. They also participate in neovascularization, essential for tissue repair and maintenance, by differentiating into vascular cells, as shown in many tissue damage models (Yamada et al., 2018; Katagiri et al., 2016; Uchida N. et al., 2017; Hosoyama et al., 2018; Fujita et al., 2021b; Hori et al., 2022).

### Presence of tissue-resident cells

Muse cells and macrophages are similar in that they both have tissue-resident cells. Muse cells are found in various tissues as SSEA-3 (+) cells, mainly in the connective tissues sporadically (Dezawa, 2016). Monocytes/macrophages are also found in tissues as tissue-resident cells, as represented by microglia in the brain, Kupffer cells in the liver, and Langerhans cells in the epidermis (Uribe-Querol and Rosales, 2020).

## The pluripotency maintenance mechanism in Muse cells differs from that in ES and iPS cells

Muse cells are pluripotent-like because they express pluripotency genes such as *NANOG*, *OCT3/4*, and *SOX2*, while at moderate levels compared with ES and iPS cells (Kuroda et al., 2010; Wakao et al., 2011); they also exhibit the ability to differentiate into ectodermal, endodermal, and mesodermal lineage cells at a single-cell level while maintaining self-renewability over generations (Kuroda et al., 2010). They secrete factors involved in self-renewal, such as AKAP13 and LXR/RXR-, FXR/RXR-, and cAMP-dependent protein kinase-pathway molecules (Alessio et al., 2017). Muse cells were shown to differentiate into various types of cells: neuronal cells, glial cells, keratinocytes, melanocytes, vascular cells, hepatocytes, cholangiocytes, cardiac cells, glomerular cells, skeletal muscle cells, adipose cells, osteocytes, chondrocytes, alveolar cells, intestinal cells, and so on, both *in vitro* and *in vivo* (Kuroda et al., 2010; Wakao et al., 2011; Yamada et al., 2018; Tsuchiyama et al., 2013; Amin et al., 2018; Katagiri et al., 2016; Uchida N. et al., 2017; Hosoyama et al., 2018; Fujita et al., 2021b; Hori et al., 2022; Uchida et al., 2016). From the broad spectrum of differentiation ability, Muse cells are considered pluripotent-like.

However, unlike ES and iPS cells, Muse cells have low telomerase activity comparable to that in somatic cells such as fibroblasts and do not show tumorigenicity when transplanted *in vivo* (Ogawa et al., 2022; Wakao et al., 2011; Kuroda et al., 2013; Ogura et al., 2014; Gimeno et al., 2017). In clinical trials, no tumorigenicity has been reported until now (Noda et al., 2020; Niizuma et al., 2023; Fujita et al., 2021a; Yamashita et al., 2023; Sato et al., 2024; Koda et al., 2024). These observations raised an essential scientific question: how do Muse cells maintain pluripotency without being tumorigenic?

Recently, the intracellular molecular mechanism of Muse cells was shown to differ from that in ES and iPS cells. In ES and iPS cells, the high expression of an oncogenic RNA-binding pluripotency-related protein, LIN28, and the absence of its antagonist, the tumor-suppressor microRNA (miRNA) let-7, serve as a key axis in maintaining pluripotency (Gunaratne, 2009). In contrast, Muse cells do not express LIN28 but only express let-7 at higher levels than iPS cells (Li et al., 2024).

The LIN28-let-7 axis works in a seesaw manner to maintain the balance between self-renewal and differentiation in ES and iPS cells (Gunaratne, 2009). LIN28 is highly expressed during early embryogenesis, but its expression decreases during development. Most somatic cells lose the expression of LIN28 after birth, and the recurrence of LIN28 expression is observed in many human cancers. Therefore, LIN28 is considered an oncogene. Let-7 is a tumor-suppressor miRNA that is highly expressed in most somatic cells, which may serve as a strategy to decrease tumorigenic risk (Gunaratne, 2009).

**FIGURE 4:**
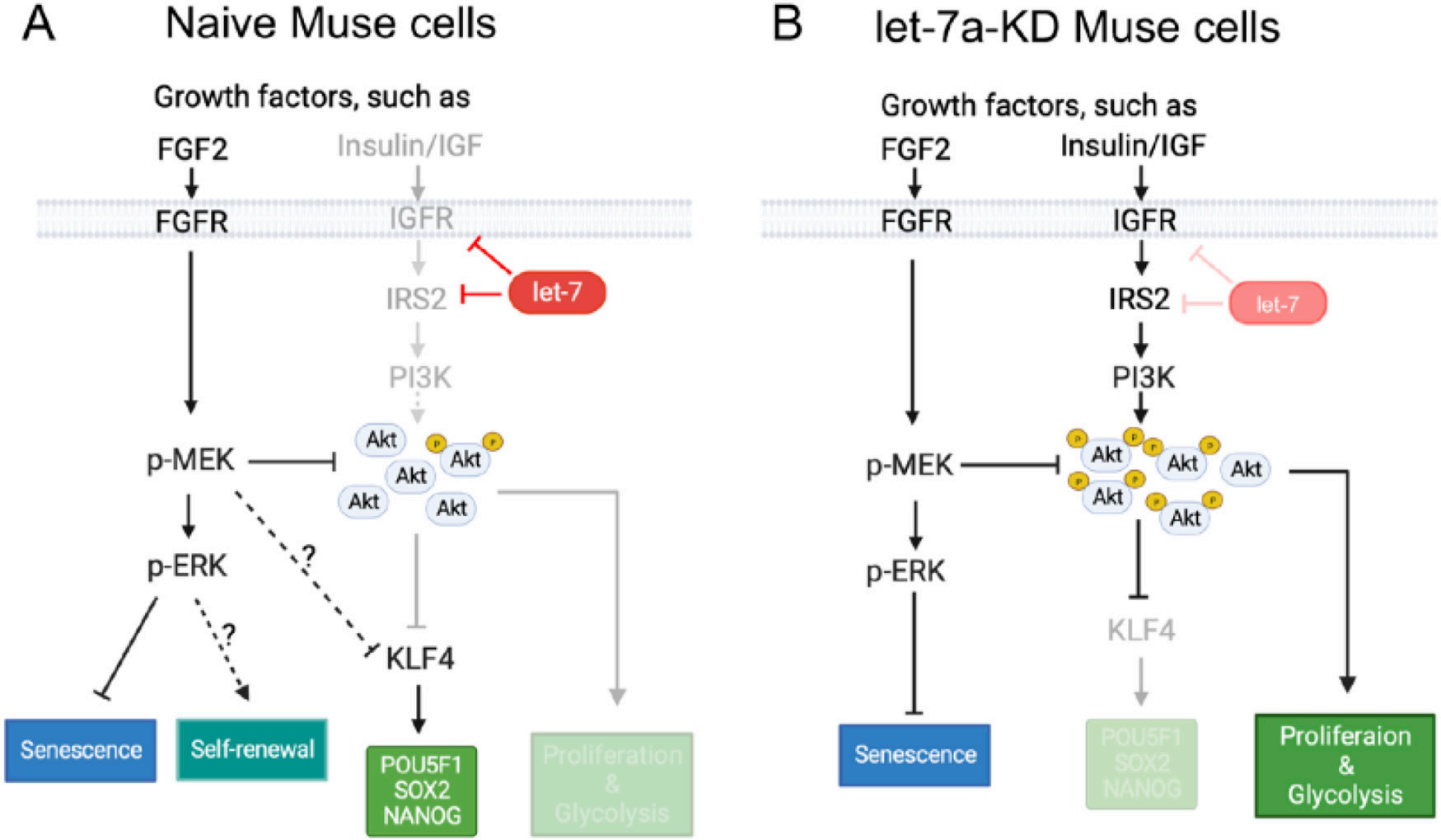
Mechanism of pluripotency maintenance in Muse cells. **(A)** In naive Muse cells, let-7 maintains the expression of pluripotency genes and inhibits proliferation and glycolysis by inhibiting the expression of IGF1R and IRS2 to repress the PI3K-AKT pathway. The PI3K-AKT pathway negatively controls the expression of *KLF4*, which promotes the expression of *POU5F1(OCT3/4)*, *SOX2*, and *NANOG*. The PI3K-AKT pathway directly inhibits proliferation and glycolysis. The MEK/ERK pathway, which seems not affected by let-7, suppresses the PI3K-AKT pathway by reducing the phosphorylation level of AKT. This pathway also inhibits the expression of *KLF4*, but it virtually does not affect the expression of pluripotency genes and is suggested to suppress senescence and maintain self-renewal of Muse cells. **(B)** Effect of let-7 knockdown (KD) on the PI3K-AKT and MEK/ERK pathways in Muse cells. The PI3K-AKT pathway is more activated than in naive Muse cells. Increased AKT phosphorylation inhibits the expression of *KLF4*, which leads to the downregulation of *POU5F1(OCT3/4)*, *SOX2*, and *NANOG*. On the other hand, cell proliferation and glycolysis were promoted. The MEK/ERK pathway reduces the phosphorylation level of AKT and also suppresses cell senescence (Li et al., 2024).

In Muse cells, let-7 was shown to be a key miRNA that maintains pluripotency through the inhibition of the PI3K-AKT pathway, leading to the sustainable expression of the key pluripotency regulator KLF4 and its downstream genes, *POU5F1*, *SOX2*, and *NANOG* (Li et al., 2024; Figure 4A). Let-7 also suppressed proliferation and glycolysis by inhibiting the PI3K-AKT pathway, suggesting its involvement in non-tumorigenicity (Li et al., 2024; Figure 4A). When let-7 was suppressed, the pluripotency and self-renewal of Muse cells were diminished, and cell proliferation and glycolysis were promoted (Li et al., 2024; Figure 4B).

The system found in Muse cells, in which the tumor suppressor let-7, but not LIN28, tunes the expression of pluripotency genes, might be a rational cell system conferring both pluripotency-like properties and a low risk for tumorigenicity.

## Muse cells differ from non-Muse MSCs, VSEL stem cells, and MIAMI cells

### Non-Muse MSCs

While Muse cells can be directly isolated from biological tissues, including BM aspirates, liposuction samples, skin biopsies, and fetal appendages such as the umbilical cord and amnion (Dezawa, 2016; Leng et al., 2019; Sato et al., 2020; Aprile et al., 2021; Ogawa et al., 2022), they can also be obtained from commercially available MSCs, comprising several percent of the total cell population (Kuroda et al., 2013). The anti-SSEA-3 antibody recognizes glycolipids, not proteins that are encoded by genes (Kuroda et al., 2013). Therefore, Muse cells have been confirmed in MSCs from multiple species, including humans, mice, rats, rabbits, swine, and goats, all as SSEA-3 (+) cells that can differentiate into triploblastic-lineage cells (Kuroda et al., 2022). The question is how the several percent of SSEA-3 (+) Muse cells compare to the 97–98% of SSEA-3 (−) non-Muse MSCs in terms of similarities and differences.

Both Muse cells and non-Muse MSCs express mesenchymal stromal cell markers, CD29, CD90, and CD105, are non-tumorigenic, and exhibit bystander effects, including anti-apoptotic, anti-inflammatory, and anti-fibrosis effects (Kuroda et al., 2013; Kuroda et al., 2022; Table 1). Unlike Muse cells, however, non-Muse MSCs differentiate only into osteocytes, chondrocytes, and adipocytes (Wakao et al., 2022; Ogura et al., 2014). They become trapped in the lung after intravenous injection, preventing them from reaching the damaged tissues, and they disappear from the body within several weeks (Yamada et al., 2018; Figure 2). Single-cell RNA sequence analysis showed the clear difference between Muse cells and non-Muse MSCs; the expression levels of the p53 repressor MDM2; signal acceptance-related genes EGF, VEGF, PDGF, WNT, TGFB, INHB, and CSF; ribosomal protein; and glycolysis and oxidative phosphorylation were higher in Muse cells than in non-Muse cells. Conversely, FGF and ANGPT, Rho family and caveolae-related genes, amino acid and cofactor metabolism, MHC class I/II, and lysosomal enzyme genes were higher in non-Muse MSCs than in Muse cells (Oguma et al., 2022).

**TABLE 1:** Comparison between tissue (BM and organ connective tissue)- and peripheral blood-Muse cells, non-Muse MSCs, VSEL stem cells, and MIAMI cells.

	Muse cells		MSCs (non-muse)	VSEL stem cells	MIAMI cells
	Tissue	Peripheral blood			
Localization					
Bone marrow	O (exist)		O	O	O
Tissues	O		O	Gonads	
Peripheral blood		O		O	
Surface markers					
SSEA-3	+	+	−		
CD29	+		+		+
CD73	+	+	+		
CD90	+		+		
CD105	+	+(low)	+		
CD45	−	+	−	−	−
CD34	−	−	−	+	−
CD31	−				
CD117	−		−		−
SSEA-4	+		−	+	+
MHC class I	+				
MHC class II	−	+			
Transcription factors					
NANOG	+	+	−	+	
OCT3/4	+	+	−	+	+
SOX2	+	+	−		
Rex1	+	+	−	+	+
Specific culture condition	Not necessary	Not necessary	Not necessary	Not necessary	Low O_2_, fibronectin
Telomerase	Somatic cell level	Somatic cell level	Somatic cell level		High

The migration of non-Muse MSCs is mainly controlled by CXCR4-SDF-1 and other axes, irrelevant to damage signals (Yang et al., 2015). On the other hand, Muse cells are pluripotent-like, differentiate into target cell types through phagocytosis *in vivo*, pass through the lung after intravenous injection, and selectively home to the damaged site via the S1P–S1P receptor 2 axis. They survive in the homed tissue as part of the tissue component for an extended period due to a specific immunotolerance mechanism (Yamada et al., 2018; Figure 2). For these differences, the bystander effect of Muse cells is occasionally long-lasting and superior to that of non-Muse MSCs, as shown in rabbit AMI, rat/mouse stroke, and hypoxic-ischemic encephalopathy, mouse chronic kidney disease, mouse hepatitis, and rat lung ischemic reperfusion models (Yamada et al., 2018; Uchida N. et al., 2017; Uchida et al., 2016; Uchida H. et al., 2017; Iseki et al., 2017; Yabuki et al., 2018). Regarding homing to the damaged site after intravenous injection, ∼15% of Muse cells are home to the site of damage, while fewer than 1% of MSCs or non-Muse cells, if any, are home to the site of damage (Yamada et al., 2018; Freyman et al., 2006; Kean et al., 2013; Figure 2).

### VSEL stem cells

Their size is reported to be 3–5 μm in mice and 5–7 μm in humans, smaller than red blood cells found in the PB, umbilical cord blood, and reproductive tissues. They are positive for SCA1, CD34, CXCR4, and SSES-1 and negative for Lin and CD45 (Kucia et al., 2006). In contrast to VSEL stem cells, Muse cells are not only found in the BM and PB but also in organ connective tissues (Dezawa, 2016; Leng et al., 2019; Sato et al., 2020; Aprile et al., 2021; Ogawa et al., 2022). Human Muse cells in the PB are 10–15 μm; thus, Muse cells are considerably larger than VSEL stem cells (Sato et al., 2020). Marker expression also differs between Muse cells and VSEL stem cells; Muse cells from the BM and organs are double-positive for SSEA-3 and CD105, whereas Muse cells in the PB are consistently double-positive for SSEA-3 and CD45. The expression of CD45 makes a sharp contrast between PB-Muse cells and VSEL stem cells. In addition, VSEL stem cells are positive for CD34, whereas Muse cells are consistently negative (Wakao et al., 2011; Table 1).

### MIAMI cells

They were found in the BM as cells positive for SSEA-4, OCT-4, and Rex1, as well as CD29, CD63, CD81, CD122, and CD164 after culturing BM cells on fibronectin at low oxygen for several weeks. They are also reported to have higher telomerase activity and were negative for CD34, CD45, CD117, and HLA-DR. MIAMI cells were shown to differentiate *in vitro* into cartilage, bone, adipose cells, and ectodermal and endodermal lineage cells. Some of the surface marker expression patterns of MIAMI cells are similar to those of BM-Muse cells. However, MIAMI cells and Muse cells differ significantly in terms of tissue distribution, marker expression, and culture methods (Table 1).

In summary, Muse cells are distinct from other somatic stem cells claimed to be pluripotent-like, such as VSEL stem cells and MIAMI cells, in terms of marker expression, size, proliferative activity, and tissue distribution (Kucia et al., 2006; D'Ippolito et al., 2004). Particularly, phagocytic activity and differentiation through phagocytosis distinguish Muse cells from these cells (Table 1).

## Reparative activity of endogenous muse cells; suggestions from clinical data

The dynamics of endogenous Muse cells in the PB of patients with ischemic stroke, AMI, and liver surgery suggested that Muse cells function as endogenous reparative stem cells (Tanaka et al., 2018; Hori et al., 2016; Kikuchi et al., 2022). Patients showed an increase in serum S1P levels before the increase in PB-Muse cells (Tanaka et al., 2018). However, Muse cell dynamics did not follow a single pattern but were primarily divided into three patterns: increase, decrease, and no change (Tanaka et al., 2018; Hori et al., 2016). An AMI study showed that PB-Muse cell dynamics in the acute phase (by day 7 after onset) played an essential role in patient prognosis; patients with a higher number of PB-Muse cells in the acute phase exhibited statistically meaningful recovery of cardiac function with a lower occurrence of heart failure in the chronic stage (6 months after onset) compared with patients who showed no increase in the number of PB-Muse cells during the acute phase. This suggested that Muse cells are involved in the innate reparative function (Tanaka et al., 2018). Similarly, patients who underwent liver surgery and showed a higher rate of increase in PB-Muse cells experienced better recovery than those with a lower rate of increase (Kikuchi et al., 2022). Thus, the number of PB-Muse cells is a potential parameter of the body’s reparative mechanisms.

In summary, endogenous Muse cells respond to the damage signal S1P, mobilize into the peripheral blood, migrate to the target tissue, and function as reparative stem cells to facilitate tissue repair (Tanaka et al., 2018). When the number of endogenous Muse cells is insufficient or their activity is impaired due to underlying conditions such as hyperglycemia or metabolic diseases, supplementing with highly active exogenous Muse cells can strengthen the body’s repair activity.

## Unique characteristics of muse cell therapy

**FIGURE 5:**
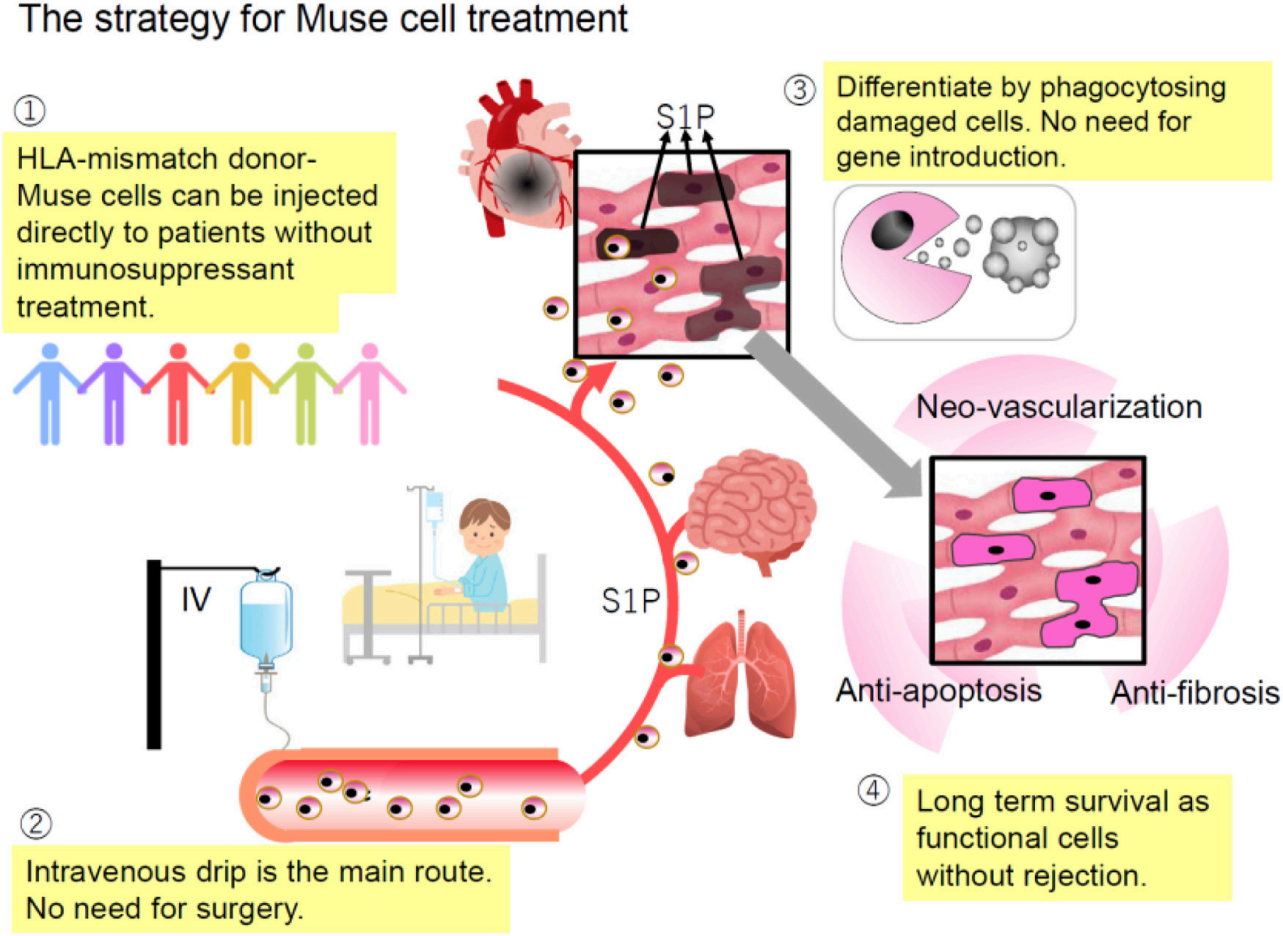
Strategy for Muse cell therapy. 1) HLA-mismatched donor Muse cells can be directly administered to patients without immunosuppressants due to specific immunotolerance; 2) since Muse cells selectively migrate to the damage site by sensing S1P produced by damaged cells, intravenous injection is a more efficient method of delivering them to the damaged site than a surgical approach; 3) unlike ES and iPS cells, Muse cells are already pluripotent-like and do not require differentiation induction because they can differentiate into the same cell type as the damaged/apoptotic cell through phagocytosis; and 4) Muse cells remain in the homed tissue for an extended period without rejection, and therefore, their bystander effects such as anti-apoptotic and anti-fibrosis effects and neovascularization are long-lasting (Minatoguchi et al., 2024).

Taking advantage of their characteristics, the strategy for Muse cell treatment can be summarized as follows (Figure 5).

1) HLA-mismatch donor Muse cells can be directly used for treatment without immunosuppression due to the specific immunosuppressive and immunotolerant mechanisms. The underlying mechanism is partly explained by the production of immunosuppressive factors related to regulatory T-cell activation, suppression of T-cell proliferation, and antigen-presenting dendritic cell differentiation, such as indoleamine 2, 3-dioxygenase (IDO), TGF-beta, PGE2, nitric oxide (NO), and HGF, as well as the expression of HLA-G, a key factor for immune tolerance in the placenta (Kuroda et al., 2022; Yamada et al., 2018; Uchida N. et al., 2017).
2) Intravenous drip is the central administration route. It does not require surgery to deliver Muse cells to the target tissue because they accurately migrate to the damaged site by sensing S1P, which is common to all tissues (Figure 2). On the other hand, MSCs respond to CXCR4 and SDF-1, which are not specific to sites of damage (Yamada et al., 2018; Iseki et al., 2017).
3) As shown by preclinical studies, ∼15% of intravenously administered Muse cells home to the site of damage, while fewer than 1% of intravenously injected MSCs home to the site of damage (Yamada et al., 2018; Freyman et al., 2006; Kean et al., 2013). Due to this high homing rate, intravenous injection of only 15 million Muse cells could produce therapeutic effects in clinical trials. MSCs generally require a 10-fold more significant number [100 – 500 million] of cells (Minatoguchi et al., 2024).
4) Gene manipulations to render pluripotency are unnecessary because Muse cells have triploblastic-lineage differentiation ability (Kuroda et al., 2010).
5) Cytokine treatment or gene introduction for differentiation induction before treatment is unnecessary because Muse cells replace damaged/apoptotic cells by phagocytosis-induced differentiation into the same cell type as the damaged/apoptotic cells with few errors *in vivo* (Wakao et al., 2022).
6) The bystander effects, such as anti-inflammatory, anti-fibrosis, anti-apoptotic, and tissue protection effects, are long-lasting because Muse cells, even allogeneic ones, survive in the host tissue as integrated cells for an extended period (Kuroda et al., 2022). They secrete various cytokines such as granulocyte colony-stimulating factor, interleukin (IL)-10, transforming growth factor-β (TGF-β), prostaglandin E2 (PGE2), vascular endothelial growth factor, platelet-derived growth factor, fibroblast growth factor, and hepatocyte growth factor and factors that suppress fibrosis such as matrix metalloproteases-1 (MMP1), MMP2, and MMP9 (Yamada et al., 2018; Uchida N. et al., 2017; Yabuki et al., 2018; Ozuru et al., 2020).
7) The low risk of tumorigenesis of Muse cells makes them beneficial for clinical applications (Ogawa et al., 2022; Wakao et al., 2011; Kuroda et al., 2013; Ogura et al., 2014; Gimeno et al., 2017).

## Clinical trials

Clinical trials for AMI, subacute ischemic stroke, epidermolysis bullosa, ALS, cervical spinal cord injury, neonatal HIE, and COVID-19 respiratory distress syndrome were all conducted via intravenous administration of donor-derived, BM-derived Muse cells without HLA matching or immunosuppressive treatment (Minatoguchi et al., 2024). This section summarizes the preclinical and clinical studies on several diseases. All the clinical trials were approved by the Clinical Trial Review Committee in the hospital and conducted in compliance with the Japanese “Act on Securing Quality, Efficacy, and Safety of Products Including Pharmaceuticals and Medical Devices,” the “Ordinance on Good Clinical Practice of Regenerative Medical Products,” and the Declaration of Helsinki. Patients (or family members, if required) provided written informed consent (Noda et al., 2020; Niizuma et al., 2023; Fujita et al., 2021a; Yamashita et al., 2023; Sato et al., 2024; Koda et al., 2024).

## AMI

### Preclinical study

The rabbit AMI model generated by ischemia-reperfusion of the coronary artery that received intravenous injections of 3 × 10^5^ autologous/allogeneic-BM-derived Muse cells (collected as SSEA-3 (+) cells from BM-MSCs) exhibited ∼14.5% engraftment into the post-infarct heart tissue (Yamada et al., 2018). In contrast, the same number of autologous/allogeneic-non-Muse cells (collected as SSEA-3 (−) cells from BM-MSCs) were mainly trapped in the lung and remained undetectable in the heart after 3 days. Engrafted Muse cells differentiated into physiologically functional cardiac cells, exhibiting Ca^2+^ influx and efflux synchronized with systole and diastole, respectively, and vascular endothelial cells. The Muse group showed a significantly reduced infarct size, improved left ventricle (LV) function, and attenuated LV remodeling compared to the non-Muse group at 2 weeks and 2 months with statistical significance. Notably, intravenously injected allogeneic-Muse cells are also differentiated into cardiac cells that could remain in the host heart tissue for 6 months without immunosuppressant treatment (Yamada et al., 2018). A similar trend was confirmed in the mini-pig AMI model, where a semi-clinical grade human Muse cell product was administered without immunosuppressants (Yamada et al., 2022).

### First-in-human clinical trial

An open-label, non-randomized, single-arm, non-controlled clinical trial was conducted in patients with ST-elevation myocardial infarction (STEMI) and a left ventricular ejection fraction (LVEF) of d≤45% after successful percutaneous coronary intervention (PCI) (Noda et al., 2020). Three patients received an intravenous drip of 1.5 × 10^7^ clinical-grade donor Muse cells for 5 days after onset. Vital signs, infectious disease tests, hematologic tests, blood biochemical tests, urinary tests, and pro-inflammatory cytokine tests (IL-1, IL-6, tumor necrosis factor [TNF]-alpha, and interferon [IFN]-gamma) were examined before and after the administration for up to 12 weeks. No blood test abnormalities were associated with Muse cell treatment. LVEF, measured by echocardiography using the modified Simpson’s method, exhibited a significant increase from 40.7% ± 1.5% before administration to 43.3% ± 2.1% at 24 h (p < 0.05), 47.3% ± 3.2% at 8 days (p < 0.001), 49.3% ± 3.2% at 2 weeks (p < 0.001), 47.7% ± 3.2% at 4 weeks (p < 0.001), 50.0% ± 3.0% at 8 weeks (p < 0.001), and 52.0% ± 2.6% at 12 weeks (p < 0.001) (12). In total, LVEF increased from 40.7% to 52.0% with the donor Muse cell treatment over 12 weeks (Noda et al., 2020). The significant increase in the LVEF by more than 10% in this clinical trial is expected to improve the long-term prognosis of AMI because the degree of LVEF recovery is suggested to predict long-term outcomes; AMI patients with no LVEF recovery (Δ ≤ 0) within 8 weeks have a higher risk of sudden cardiac arrest and death compared to those with a modest increase (Δ1% to 9%) or a significant increase (Δ ≥ 10%) in the LVEF over a 5-year follow-up period (Chew et al., 2018).

## Epidermolysis bullosa

### Preclinical study

Epidermolysis bullosa (EB) is a group of genodermatoses characterized by widespread blisters and erosions caused by mutations in the basement membrane zone (BMZ) genes. EB is roughly categorized into EB simplex (EBS), junctional EB (JEB), and dystrophic EB (DEB) (Fine et al., 1991). Type XVII collagen-knockout (Col17-KO) mice simulate JEB in which few animals can survive for an extended period (Nishie et al., 2007; Fujita et al., 2010). Adult Col17-KO mice received intravenous injection of 5.0 × 10^4^ Nano-lantern-labeled human BM-Muse and -non-Muse cells three times a week every 2 weeks without immunosuppressants (Fujita et al., 2021b). Muse cells homed to skin injury sites more effectively than non-Muse cells, with statistical significance, and differentiated into keratinocytes in the epidermis and hair follicle cells, vascular endothelial cells, and sebaceous glands in the dermis. Notably, all the mice that received Muse cells showed a linear deposition of human-type VII COL (hCOL7) and -Col17 in the BMZ of the injury site (Fujita et al., 2021b). When Col17-KO mice received a single intravenous injection of 3 × 10^5^ cells/body (corresponding to 3 × 10^7^/kg) of clinical-grade human Muse cell preparation, they showed rapid wound healing, slower expansion of the affected area, and sustained deposition of hCOL7 and hCOL17 in the BMZ for more than 6 months without immunosuppressants. Interestingly, hair loss and the development of gray hair were substantially suppressed in the treated mice (Fujita et al., 2021b).

### Clinical trial

A phase I/II open-label, non-randomized, single-arm, non-controlled clinical trial was conducted (Fujita et al., 2021a). Five adult DEB patients (one man and four women, 26.8 ± 12.8 years old) with 13 refractory/recurrent ulcers were enrolled. A single dose of the clinical-grade donor-Muse cells [1.5 × 10^7^ cells (2.98 ± 0.61 × 10^5^ cells/kg)] was intravenously administered, and patients were followed for 52 weeks. Two patients showed a >50% reduction per patient in the area of the selected ulcer at 4 weeks after treatment. Overall, the combined size of the selected ulcers was significantly reduced during the 52-week observation period, with reduced pain in the ulcer area and improved serum liver enzymes, possibly due to the anti-inflammatory effect. No prominent adverse events associated with the treatment were reported, except for one patient who experienced transient paresthesia (Fujita et al., 2021a). Further clinical trials with multiple infusions of Muse cells are expected to deliver more significant therapeutic effects with long-term safety.

## Ischemic stroke

### Preclinical study

Intravenous injection of human BM-Muse cells into a mouse lacunar stroke model on days 9 (subacute phase) and 30 (chronic phase) exhibited that injected Muse cells reached the lacunar infarction area by day 1 (Abe et al., 2020). Muse cells in the post-infarct region started to express early neural markers, Mash1 and NeuroD, on day 3 and maturity markers, MAP2 and NeuN, on day 7, making connections (Uchida et al., 2016; Uchida H. et al., 2017). Muse cell-derived neuronal cells were incorporated into the pyramidal and sensory tracts, as shown by the recovery of somatosensory-evoked potentials and the formation of synapses with host neuronal cells, leading to statistically meaningful therapeutic effects in motor (modified neurologic severity score and rotarod, corner turn, and cylinder tests) and sensory functions by 3 months (Uchida et al., 2016; Uchida H. et al., 2017). The extent of functional recovery was dose-dependent and effective even when the injection was administered on day 30 (Abe et al., 2020).

When integrated Muse cells were eliminated at 8 weeks through a loss-of-function experiment by administrating diphtheria toxin, functional recovery remained stable, but active recovery was abolished and dropped sharply to the vehicle group level, suggesting that integrated Muse cells mediated the behavioral outcome (Uchida H. et al., 2017).

### Clinical study

A double-blind, placebo-controlled clinical trial enrolled ischemic stroke patients with a modified Rankin Scale (mRS) ≥3 (Niizuma et al., 2023). Randomized patients received a single-dose intravenous injection of either the clinical-grade donor Muse cells (1.5 × 10^7^ cells) (n = 25) or placebo (n = 10) without immunosuppressants, 14–28 days after onset. Safety (primary endpoint: 12 weeks) and efficacy (mRS, other stroke-specific measures) were assessed until 52 weeks. The key efficacy endpoint was the response rate (percent of patients with mRS ≤2 at 12 weeks). The response rate was 40.0% (95% CI, 21.1–61.3) in the Muse cell group and 10.0% (0.3–44.5) in the placebo group; the lower CI in the Muse cell group exceeded the preset efficacy threshold (8.7% from registry data) (Niizuma et al., 2023).

Significant improvements in the Fugl-Meyer Motor Scale upper limb and total scores were observed as early as 4 weeks and continued up to 52 weeks (Niizuma et al., 2023). Early improvements in the upper limb function are generally difficult to achieve and directly related to improving mRS to 0 or 1. Interestingly, in the Muse cell group, 12% of patients experienced a change in hair color from grey/white to black within 12 weeks, and 24% of patients experienced a change in hair color between 12 weeks and 52 weeks, while no patients in the placebo group experienced such a hair color change (Niizuma et al., 2023).

## ALS

### Preclinical study

SOD1-G93A transgenic mice that expressed the G93A mutant form human SOD1, known to exhibit a phenotype similar to that of ALS patients, were used (Gurney et al., 1994). Human BM-Muse cells (5 × 10^4^ cells) were intravenously injected starting on day 56 after birth and continued once a week until 119 days, for a total of 10 injections (Yamashita et al., 2020). Following intravenous administration, Muse cells homed to the cervical and lumbar spinal cord, important therapeutic targets for ALS, within 7 days. The therapeutic effects in the Muse cell group were superior in the behavioral test scores, motor neuron survival, and reduced myofiber atrophy than in the MSC-treated or vehicle groups (Yamashita et al., 2020).

### Clinical trial

Unlike in the clinical trials for ischemic stroke, in which tissue damage occurs suddenly, motor neuron degeneration progresses slowly and continuously in ALS. Therefore, in an open-label, non-randomized, single-arm, non-controlled clinical trial, the clinical-grade donor Muse cells (1.5 × 10^7^ cells) were administered multiple times (once a month for six doses without immunosuppressants). Five patients with sporadic ALS (male-to-female ratio of 3:2) with the limb-onset clinical form were enrolled (Yamashita et al., 2023). The primary endpoints were safety and tolerability, and the secondary endpoint was the rate of change in the Revised Amyotrophic Lateral Sclerosis Functional Rating Scale (ALSFRS-R) score for up to 12 months.

Despite repeated administration of Muse cells from donors without HLA matching or immunosuppressants, the treatment was well-tolerated, with no reported cases of pulmonary embolism, anaphylactic shock, or other serious adverse effects. ALSFRS-R scores remained unchanged for four patients and showed a trend toward exacerbation for one patient for up to 10 months after initiating cell administration, strongly suggesting the therapeutic potential of donor Muse cell treatment in mitigating ALS progression. Unfortunately, 12 months after the first dose, one patient fractured the proximal left humerus and left ankle joint during a fall. This incidental adverse event worsened clinical scores at 12 months, resulting in the ALSFRS-R scores tending to improve 12 months post-treatment compared with 3 months before Muse cell administration, while the difference did not reach statistical significance, likely due to the incident in one patient (Yamashita et al., 2023).

## Neonatal HIE

### Preclinical study

Seven-day-old HIE model rats received human BM-Muse cell intravenous injection 72 h after onset without immunosuppressants (Suzuki et al., 2021). Human Muse cells were distributed mainly to injured regions in the brain at 2 and 4 weeks, differentiated into neuronal and glial cells. They remained in the brain tissue as neural cells for 6 months without immunosuppressants. In contrast, non-Muse BM-MSCs were mainly trapped in the lung at 2 weeks and disappeared from the body by 4 weeks. Magnetic resonance spectroscopy and positron emission tomography showed that Muse cells dampened excitotoxic brain glutamatergic metabolites and suppressed microglial activation, significantly improving motor and cognitive functions at 4 weeks and 5 months (Suzuki et al., 2021).

### Clinical trial

A single-center open-label dose-escalation clinical trial was conducted with nine moderate-to-severe HIE neonates who received therapeutic hypothermia (Sato et al., 2024). Each patient received a single-dose cell intravenous injection of donor Muse cells between 5 and 14 days of age. The low-dose group (three patients) and high-dose group (six patients) received 1.5 × 10^6^ and 1.5 × 10^7^ cells/dose, respectively. No significant changes in physiological signs, including heart rate, blood pressure, and oxygen saturation, were observed during or after administration, and the only adverse event was a mild γ-glutamyl transferase level elevation in one neonate, which spontaneously resolved without any treatment. All patients survived, and normal developmental quotients (≥85) in all three domains of the Kyoto Scale of Psychological Development 2001 were recognized in 67% of the patients (Sato et al., 2024). Donor Muse cell administration was demonstrated to be safe and tolerable for neonates with HIE (Sato et al., 2024). A randomized controlled confirmatory study will warrant the efficacy of this therapy.

## Spinal cord injury

### Preclinical study

Human clinical-grade Muse cells were intravenously administered to the rat acute (1 day) and subacute (2 weeks) mid-thoracic spinal cord contusion models (Kajitani et al., 2021; Takahashi et al., 2023). Hind limb motor functions significantly improved from 14 to 56 days in the acute model and 6 to 20 weeks in the subacute model, with statistical significance to the vehicle group, respectively. In both models, the cystic cavity became smaller. More significant numbers of descending 5-HT fibers were preserved in the distal spinal cord. Human BM-Muse cells were differentiated into neuronal and glial cells in the injured gray and white matter of the spinal cord. For the loss-of-function study that selectively ablates human cell functions in the subacute model, diphtheria toxin was administered intraperitoneally to animals at 20 weeks, which resulted in the quick cancellation of the functional improvements provided by Muse cells, suggesting that engrafted Muse cells contributed to the functional recovery (Kajitani et al., 2021; Takahashi et al., 2023).

### Clinical trial

A prospective, multicenter, non-randomized, non-blinded, single-arm study was conducted in cervical spinal cord injury patients with a neurological level of injury C4 to C7 that showed the severity of modified Frankel classification B1 and B2 (Koda et al., 2024). The present clinical trial recruited 10 participants (eight male individuals and two female individuals) with an average age of 49.3 ± 21.2 years. All 10 participants received a single dose of donor Muse cells (1.5 × 10^7^ cells)(2.1–2.7 × 10^5^ cells/kg of body weight) by an intravenous drip. The ISNCSCI motor score, the activity of daily living, and quality of life scores after treatment showed statistically significant improvements compared to those before administration (Koda et al., 2024).

## Future perspectives

Various types of stem cells exist in the body. Muse cells have unique characteristics not observed in other stem cells. They can be identified as cells positive for the pluripotent surface marker SSEA-3, show pluripotent- and macrophage-like properties, and phagocytosing dead cells differentiate into the same cell type as the phagocytosed cell. They are suggested to contribute to tissue homeostasis by minute tissue repair through phagocytosis-induced differentiation and cell replacement. Their therapeutic effects in clinical trials by donor Muse cell intravenous injection without immunosuppressant treatment have been reported. ^5^Clinical trials were all conducted by intravenous drip of allogeneic-human BM-Muse cell product without immunosuppressant treatment. Tumorigenesis or severe side effects that required interruption of the trials have not been reported during the follow-up (Noda et al., 2020; Niizuma et al., 2023; Fujita et al., 2021a; Yamashita et al., 2023; Sato et al., 2024; Koda et al., 2024). Donor cells have several advantages over autologous cells because donor cells are ready to use for emergency cases and acute phases, are less burdensome for the patients than collecting autologous Muse cells, and quality variation is easy to control. The loss-of-function experiment in the lacunar stroke and spinal cord injury rodent models revealed that eliminating Muse cells that were once integrated as neural cells rapidly abolished functional improvement (Uchida H. et al., 2017; Kajitani et al., 2021). In the same manner, functional improvement provided by integrated Muse cells will diminish or disappear if the cells are rejected due to an immunologic response in clinical trials. In the clinical trial of ischemic stroke, statistically meaningful functional recovery was maintained for up to 52 weeks in the Muse cell-treated group, suggesting that donor Muse cells were unlikely to be rejected until that time point (Niizuma et al., 2023). The mechanism underlying the long-term survival of HLA-mismatched Muse cells in host tissue must be clarified in future studies, and future investigations should include placebo-controlled, double-blind studies with a large population in each clinical trial to enhance verification.

The clinical studies revealed that the optimal conditions for administration timing, number of administrations, and number of donor Muse cells varied depending on the disease pathology. In acute tissue damages such as in AMI and stroke, a single dose was therapeutically effective (Noda et al., 2020; Niizuma et al., 2023). However, a different approach may be needed for chronic diseases such as ALS and EB. In ALS clinical trials, multiple doses of donor-Muse cells prevented or slowed the deterioration of the disease condition (Yamashita et al., 2023). However, in EB, where only one dose was administered, improvement was confined to a certain period (Fujita et al., 2021a), suggesting that multiple doses could produce more potent therapeutic effects. In summary, the optimal conditions for acute injury and chronic disease differ in timing and number of administrations.

Unlike Muse cells, conventional MSCs applied in many clinical studies can only differentiate into confined cell types, chondrocytes, osteocytes, and adipocytes (Ogawa et al., 2022; Wakao et al., 2011; Kuroda et al., 2013; Ogura et al., 2014; Gimeno et al., 2017), and their therapeutic effects largely depend on bystander effects rather than cell replacement (Fu et al., 2017). Non-Muse MSCs and MSCs do not efficiently reach damaged tissues but become trapped in the lung capillaries and disappear from the body within a couple of weeks due to rejection (Yamada et al., 2018). Since there was no enrollment of an MSC group in Muse cell clinical studies, the therapeutic effects of Muse cells and MSCs cannot be directly compared. However, in animal experiments, a comparison of the therapeutic results between Muse and MSC/non-Muse cells consistently demonstrated the superiority of Muse cells over MSC/non-Muse cells in AMI, stroke, aortic aneurysm, peripheral artery disease, neonatal hypoxic-ischemic encephalopathy, spinal cord injury, ALS, chronic kidney disease, liver injury, and skin disease models (Yamada et al., 2018; Uchida N. et al., 2017; Hosoyama et al., 2018; Fujita et al., 2021b; Hori et al., 2022; Uchida H. et al., 2017; Yamashita et al., 2020; Suzuki et al., 2021; Kajitani et al., 2021; Shimamura et al., 2017; Fei et al., 2021; Ueda et al., 2023). The differences in those outcomes are suggested to be due to the difference in tissue repair mechanisms between Muse cells and MSCs.

Muse cells constitute a small percentage of MSCs. Even when MSCs are administered into the blood, which is comprised of large amounts of non-Muse cells and a small number of Muse cells, Muse cells cannot bring about a repair effect in the body. This is suggested due to the anti-inflammatory effect of non-Muse cells, which suppresses the production of S1P that should be released from the damaged site, making it difficult for Muse cells to find the damaged site (Takahashi et al., 2024).

Muse cells are naturally existing endogenous reparative stem cells with both pluripotent- and macrophage-like properties; they may provide a next-generation cell therapy that does not rely on artificial gene introduction or manipulation to treat a wide range of diseases.

## Is there any hierarchy?

Since Muse cells, MSCs, VSEL stem cells, and MIAMI cells are collected from tissues, there is an ongoing discussion regarding their relationship and hierarchical organization (Aprile et al., 2024; Cancedda and Mastrogiacomo, 2024).

Like hematopoietic stem cells, the main reserve of Muse cells is considered the bone marrow. Muse cells show characteristics similar to monocytes/macrophages, immunological cells that belong to the hematopoietic lineage (Kuroda et al., 2010; Wakao et al., 2022). They are also found in the extraembryonic tissues such as the umbilical cord and amnion (Leng et al., 2019; Ogawa et al., 2022; Kushida et al., 2024). Based on these findings, they may originate from the extraembryonic mesoderm. Determining whether Muse cells share the same origin as macrophages is an intriguing question. However, this hypothesis should be carefully interpreted through cell lineage tracing studies.

Like other somatic stem cells, Muse cells randomly undergo symmetric and asymmetric division (Wakao et al., 2018). Therefore, when they are 100% purified and expanded in adherent culture, non-Muse cells are produced through asymmetric division, causing the proportion of Muse cells to decline rapidly and then plateau at a few percent, similar to MSCs (Wakao et al., 2018). This repeatable cycle across generations—Muse cell purification, expansion in culture, decrease in Muse cell proportion, return to MSCs, and subsequent Muse cell purification—suggests that Muse cells are genuine stem cells within MSCs and occupy the top tier in the hierarchy of heterogeneous MSCs.
